# ‘To do or not to do’? The neurobiology of decision-making in daily life: I. Getting the basics

**DOI:** 10.1590/S1980-57642008DN10100003

**Published:** 2007

**Authors:** André Palmini, Victor Geraldi Haase

**Affiliations:** 1 MD, PhD, Department of Internal Medicine, Division of Neurology, Faculty of Medicine, Pontifícia Universidade Católica do Rio Grande do Sul (PUCRS) & Neurology Service, Hospital São Lucas da PUCRS, Porto Alegre, Brazil.; 2 MD, PhD, Department of Psychology, Universidade Federal de Minas Gerais, Belo Horizonte, Brazil.

**Keywords:** decision making, executive functions, impulse control, frontal lobes, reward, tomada de decisões, funções executivas, controle de impulsos, lobos frontais, recompensa

## Abstract

The constant conflict between decisions leading to immediate pleasurable consequences versus behaviors aiming at long-term social advantages is reviewed here in the framework of the evolutionary systems regulating behavior. The inescapable temporal perspective in decision-making in everyday life is highlighted and integrated with the role of the executive functions in the modulation of subcortical systems. In particular, the representations of the ‘non-existent’ future in the prefrontal cortical regions and how these representations can bridge theory and practice in everyday life are addressed. Relevant discussions regarding the battle between emotions and reasons in the determination of more complex decisions in the realm of neuroeconomics and in moral issues have been reserved for a second essay.

## Abstract (pt)

O conflito constante entre decisões que levam a conseqüências prazerosas imediatas versus comportamentos direcionados a vantagens sociais no médio e longo prazo é aqui revisado sob o prisma de sistemas neurobiológicos que evoluíram ao longo da escala filogenética para regular o comportamento. O artigo enfatiza a inescapável perspectiva temporal na tomada de decisões na vida cotidiana, integrando-a com o papel das funções executivas na modulação de sistemas subcorticais. Em particular, as representações no cortex préfrontal de um futuro que, em realidade, ‘não-existe’ em termos concretos são conceptualizadas como a ligação entre a teoria e a prática nas questões do dia-a-dia. Discussões relevantes em relação à ‘batalha’ entre razão e emoção na determinação de decisões mais complexas nos campos da neuroeconomia e das questões morais foram reservadas para um segundo ensaio.

## We, special animals

Reaching the acumen of the phylogenetic scale came at a price and this was the need for our human brain to adapt to the demands imposed by a life-in-society. A major trade off of life in human societies is the need to shape behavior to accommodate rules, laws, and social contexts in general. It is indeed a trade-off, because such adaptations more often than not run in clear opposition to our more basic and pleasurable impulses and biological instincts. And these instincts that we share with other animals – along with the neural systems organizing them - have preserved life on earth for millions of years^1-2^. Now, the potentially bad news – at least as far as our decision- making in everyday life goes – is that this very humane need to calibrate behavior toward impulses and instincts *is not* accompanied by a reduced cerebral representation of these very instincts.

Actually, while on the one hand evolution led to the development of structures and circuits which are able to control and modulate the activity of phylogenetically older structures and systems, on the other these more ancient structures carry on working at full speed, and the conflicts thus generated are, as we will see, inevitable. Being alive and awake signifies being constantly bombarded by internal or external stimuli. We are always being reminded of our basic biological needs, we ‘feel’ our viscerae, we see, hear, touch, taste, and smell the world, and our minds are constantly generating ideas that could also be seen as ‘wishes’. All these are stimuli demanding reactions, that is, behaviors. Indeed, for each stimulus we receive – and we receive stimuli constantly – our brain generates a behavior, and each behavior will lead to a ‘consequence’. According to the consequence – that is, the result *in the future* of our reaction toward a specific stimulus – we will experience positive or negative feelings at the mental, somatic, or usually both levels. Was it advantageous having decided in that direction? Or did the consequence bear the mark of a sour punishment?

Obviously, each cycle ‘stimulus-response – consequence – affective value of the consequence’ then leads to new behaviors, new social interactions, new worries, new responses, and so on.

Even if theoretically there are a number of response options to the majority of stimuli we are constantly faced with, it is important to realize that whatever response we choose will have opposing consequences in the temporal dimension: one in immediate and another in future terms^3^. This, of course, poses the significant dilemma of making decisions based on the immediate consequence or on that expected in the future. There is no way out. Either the decision will lead to an immediate reward, without overly considering the future consequences of that act, or the behavior is geared toward a future gratification (or evitation of punishment), thus giving up the prospect of an immediate reward. Naturally, this essential dilemma of human life has been the focus of many literary, philosophical, and psychological studies. More recently, the neurosciences have decided to join the fray.

This essay presents a framework of the ways the human brain has adapted in order to perceive, analyze, and respond to stimuli, usually (that is, when the brain is healthy) aiming to achieve the most favorable consequences for a given person at a specific point in time.We will be discussing the contexts and the social consequences of human decisions, attempting an integration of the neurobiological view with the more prevalent, psychological (moral) view of what determines – or should determine – the way humans decide. In a second essay, to appear later, we will provide an in-depth discussion of the battle between emotions and reasons in the determination of more complex decisions in the realm of both neuroeconomics and moral issues.

## Decisions: the concepts of time and future and how they relate to the prosaic side of life

Regardless of whether we are talking about your beloved friend or your neighbor’s dog, each behavior is going to become part of a chain of events beginning with a stimulus (internally generated or externally provided) that will lead to a response (a motor act to approach something or someone, to run away, to say something, to do something, to do something else, etc). In turn, this response will lead to a consequence that will be felt, experienced, in the future. Of course, ‘future’ can be 10 seconds or 10 years later, but what is key is that each act we and our fellow animals perform now will have a *future* consequence. However, there is an enormous difference between *the concept of future* for a non-human animal and for a human being. Animals usually respond to the environment in an impulsive and instinctive fashion, centered in the present and hoping for advantageous consequences only in the immediate future^1-2^. To predate is to satisfy the hunger felt now. To flee a predator is to save life, now. Thus, the respective actions of aggression and defense are intended to achieve immediate results, paramount to fulfill needs as basic as feeding or surviving the dangers of the moment. There is no room to ponder over long- term consequences. In this context, the idea of the future is always the next moment, and the perspective of this immediate future as a determinant of behavior is both instinctual and conditioned by experience or genetics. With human beings, however, the story is much more complex.

In humans, the time component is crucial to the orchestration of behavior and we suggest that it is the strongest contributor to the dilemma of decision-making we all constantly face^4^.We invite the reader to consider two points in time – or two ‘times’. The first is the variable time between any given stimulus and the response to that stimulus; let us name it ‘*time 1*’. In addition, there is the time between the response, i.e., the decision or the behavior in relation to the stimulus and the consequence of that decision. I suggest referring to the latter as ‘*time 2*’. We will refer to these ‘times’ below.

The responses we provide to the stimuli we constantly receive may be more or less impulsive. The more impulsive they are, the more they target the satisfaction of the needs or wishes for which we want an *immediate reward.* However, the complex side of the ‘time component’ in the equation is that oftentimes an immediate reward is inevitably linked to some kind of punishment (in a broader sense) in the long term. Thus, the consequences of our acts provide a continuous feed-back in the decision making process and, in normal conditions, this leads to a progressive maturation of a person’s capacity to decide with a view to positive consequences within the social context of that particular individual under those particular circumstances^5^. Achieving this latter goal often implies a less positive or even an overtly negative immediate consequence, but a much larger reward in the future.

The real theme behind this ‘time’ story, and the one we invite the reader to reflect upon, is that whereas we constantly need to decide, and these decisions will impact our future, this future does not in fact exist. This is the really fascinating aspect of it all: our brain orchestrates our decisions, i.e., regulates our behavior targeting positive consequences to be enjoyed at a time that *does not exist*. In practice, the future is but a hypothetical concept, or a neural representation. As with many other important concepts, this one is better explained and perhaps understood by bridging neuroscience with the prosaic side of life.

Let us think of the following, admittedly common, scenario. This beautiful young woman is well beyond her ideal weight and decides, in a given month of April, that she will do her best to lose weight, exercise, and thus reduce the size of her bikini for the following Brazilian summer. In the period of time which separate this April from the next January, this woman will face, everyday, a challenge to her ‘alimentary behavior’ if you will. In all meals she will be stimulated visually, olfactorily, as well as interoceptively (that is the sensation of hunger and the secretions of her digestive apparatus) and will need to respond to these stimuli in one way or another. Practically speaking, she may always have the option to satiate her hunger with, for instance, those marvelous chocolate cherry cakes that would give her an enormous pleasure ‘now’, but would certainly not contribute to the bikinireducing long-term goal. On other occasions, she will be tired, very much wishing to stay relaxed at home, but will have to decide between this more relaxed approach versus going out for one hour of exercise. Again, the relaxing pleasure *now*, opposing the sacrifice for the bikini-reducing *future* goal. The crucial detail of these prosaic options is that the reward or pleasure derived from the chocolate cherry cake or the relaxation at home is immediate, real, physically enjoyable. On the other hand, refraining from these immediate pleasures, controlling the impulse to enjoy the ‘delices’ at her disposal *now*, is to decide in favor of a delayed reward, in a hypothetical future that exists only in her mental representation of how next Summer is going to be. In common parlance, this is trading what is certain for what is doubtful!

If this woman’s tale is regarded as too prosaic and perhaps undeserving of these pages, the reader could then picture another more serious, yet relatively common scenario. Consider people who follow religions, or more to the point, the proposals of every religion, irrespective of which: always trading the certain for the doubtful. In other words, the proposal is clear: you refrain from pleasures that you could get *now*, you engage in actions that are unrewarding *now* – such as sacrifices, for instance –, in exchange for a great(er) retribution in the future, in another life, in paradise, etc, depending on the religion itself. In fact, there is probably nothing as dependent upon the representation of a hypothetical future for each individual than the notion of faith and what that notion entertains in terms of behavior. Just think about the concepts of sacrifice and abstinence. These are explicit decisions based upon a moment in time – i.e., the future - that does not exist except in the mind of the believer. And these decisions, or this faith in the future are so intense and taken so seriously that they lead, by themselves, to sensations of well being, also known as peace of mind.

Inescapably, making decisions is a constant demand upon our brains, and there is always the dichotomization between the more immediate rewards and the more delayed gratifications (without the immediate rewards). If you drive and have ever been in a hurry to arrive on time at a particular place, you have had to decide - even if you did not consciously realized it – between driving faster and obtaining the ‘reward’ of avoiding being late for an appointment (but risking a fine or an accident), versus driving at the recommended speed, accepting the ‘punishment’ of arriving late, but not risking paying the expensive fine a few weeks later. And so on, indefinitely, in all spheres of our lives.

Some decisions are easier, others more difficult and some terribly anxiogenic.Most will be made in an automatic, pre-conscious fashion, and others will result from intense reflexion. It does not matter, the dilemma is always the same, and the players, immediate versus distant future, are always there. And all this as a result of a unique socio-biological combination: we have an extensive repertoire of behavioral options, while at the same time we are constrained by the fact that we are social beings. Therefore, as social beings we need to be flexible about our decisions, and this is not irrelevant: a given attitude may be entirely fitting for a given circumstance while dangerously inappropriate for another. Being able to freely decide among a range of behaviors while at the same time being constrained by the impact of each behavior in the individual’s social context in the shortand in the long-term, is perhaps the best synthesis of the interaction between the human brain and its environ-ment. Furthermore, it provides indisputable evidence of the uniqueness of each human being, no matter how many billion we number.

## Executive functions and the music of decisions

It is time to revisit the concepts of ‘time 1’ and ‘time 2’ which were outlined above. Let us consider ‘time 1’, i.e., the time between receiving a stimulus and responding to this stimulus. Let us also bring back the prosaic real life situations we analyzed: the moment the chocolate cherry cake arrives at the table at the end of the young woman’s meal, or the moment the opportunity crosses the mind of our religious friend of obtaining an advantage through illegal means, or even that moment when you look at your watch and realize you are going to be late for that most important of meetings. At this exact moment, ‘time 1’ gets going, that is, in a few minutes, days or seconds, respectively, the person will need to make a decision. As soon as the decision is made, and a response (behavior) is generated - and for all purposes let us concede that all decisions were made in the same vein, i.e., the woman declined the dessert, your friend changed his mind about the wrongdoing and you decided not to speed in your car – ‘ time 1’ is over and ‘time 2’ kicks in. In other words, following the decision, it is time to wait for the consequences, which is the definition of ‘time 2’: the period between the response and the experience of the consequence of that behavior. Let us now functionally dissect what is going on in the brain during ‘time 1’, i.e., while the stimulus is being analyzed and the decision is *in the process* of being made.

We denote as executive functions the group of functions the brain has at its disposal to analyze incoming stimuli, to rate these stimuli in regard to the individual context, and devise behavioral reactions toward them^3,6-8^. In explaining these executive functions and their role in decision-making we can draw on a prosaic comparison, that of an artist playing an accordion. The music thus produced is fully dependent upon the expansions and contractions of the middle section of the accordion. If you can keep this image in your mind while the discussion continues, an easier understanding of the executive functions and the ways the brain manages to make decisions can be grasped.

As discussed above, a healthy decision-making process should provide for advantageous decisions in the social context, which usually means in the middle to long-term future. Thus, the brain has to *compare* each new stimulus with (a) similar situations experienced in the past or transmitted through culture; (b) similar decisions made in the past when faced with a similar stimulus in a similar context, or the same as transmitted through culture; (c) cognitive or intellectual remembrances regarding the consequences of those decisions; and (d) affective or emotional memories regarding how those consequences ‘felt’ (in somatic terms). Furthermore, the brain has to ‘look into the future’ and anticipate, based upon previous personal or cultural experiences, the most likely scenarios of the potential future consequences linked to the range of options available as decisions^9-12^.

And here enters the accordion. Before we make any decisions, that is, behave in relation to a stimulus, our brain ‘looks’ into the past (the accordion opens to one side) at the same time that it ‘looks into the future’ (the accordion opens to the other side). Depending on the type of decision and the elements involved in that decision (the ‘music’), the accordion will open more to one side or to the other. However, it is certain that for the music to be played correctly, the accordion will have to open to both sides. In other words, there is no way of deciding in a correct and contextualized way in the present, without references to both the past and the foreseeable future^4,10^. And this very humane ability has progressed through the anatomical growth and specialization of the prefrontal regions, which evolved to be able to represent the present, the past, and the future at any given time, as if the three time dimensions could be woven into one, and to then integrate the necessary elements for a correct decision based on this ‘unity’. The bridges between these temporal dimensions, crucial for decisionmaking, constitute the executive functions.

## Executive function number #1:behavioral inhibition

Making decisions while taking into account past and future scenarios, is not possible through impulsive reactions to stimuli. In other words, decisions are not adequately made if ‘time 1’ is too short, and this is exactly what happens when impulsive decisions are made^3^. Often, impulsive behaviors are related to habit formation and thus, switching the pattern of decision in specific circumstances – when alternative behaviors become the best choice – demands impulse inhibition. This is commonly the case when circumstances change and behaviors that used to lead to rewards begin to lead in-stead to punishments^11^.

Prosaic examples abound. Imagine, for instance, an affective relationship recently over. A series of ‘automatic acts’ in relation to the ex-partner become inadequate and have to be refrained from. Behaviors such as phone calls and physical approaches may then lead to disappointment or punishments the following day or week, for instance. Under the new circumstances, a behavior previously associated with pleasure may now lead to punishment in some form. Thus, the impulse of acting as before has to be contained and give way to reflecting about the consequences in the near future. Behavioral inhibition becomes crucial.

## Executive function number 2: working memory, or what memories are really for

‘Working memory’ could be conceived as the executive function which activates our episodic and affective memories into practical use. Of course, it is good to have episodic remembrances of events we have experienced, rock concerts, soccer matches, elections, and so forth. And also of what happened yesterday and the day before which give our lives a meaningful sequence in time. However, we suggest that the major utility of our memories is to help craft our decision-making in the present. Working memory, thus, is the function through which the brain brings to the fore those specific memories relating to the specific stimuli we are facing in the present, and to which we have to devise a response. In other words, it is the function that recalls to the present, for short periods of time, a set of memories that are stored throughout the brain and which have in common the fact they apply to the decision to be made. Furthermore, this rapid recovery evokes the affective state generated by the consequences of those decisions back then, and provides a framework of what we may expect in the future should behavior *a, b*, or *c* be the chosen. By bringing to the present what happened and how we felt in the past in similar situations, working memory allows the development of behaviors with a greater likelihood of a favorable outcome^4,6,10,12^. Common parlance refers to this as a process of behavioral maturation resulting from experience. Poor working memory function implies dealing with only short-term perspectives, in a sense, being a ‘prisoner of the present’. The ‘orchestration of behavior in time’10 is compromised because the ability to integrate present/past/future in relation to a stimulus and the possible responses to that stimulus is defective*.

An alternative way to illustrate what working memory does is to picture a mental blackboard on which only the set of memories relevant for the decision in question is written and, and once the decision is made, is immediately erased to allow space for the next issue, and so on. The majority of these memories are probably recalled in a preconscious fashion, largely as cerebral representations, but serve the purpose of preselecting the most favorable options.

## The balance between pleasure versus social obligations: the reward system of the brain comes into play

Studies on primates, human lesions, and functional imaging have demonstrated that the prefrontal regions are key to the organization of executive functions and thus to decision-making. Clinically, prefrontal lesions do interfere with executive functioning and the ability to decide based on the current contextual circumstances^3,6- 8,10,12,15-23^. Details on the phylogenetic and ontogenetic evolution of the frontal lobes and further details on its anatomofunctional organization in regard to executive functions and decision-making in general, constitute a major issue in itself and will not be further dealt with here for obvious reasons of space. At this point, however, we would like to introduce a subcortical system which constantly defies the prefrontal regions.

Evolution has not deleted subcortical structures key to animal and species survival, but has progressively added cortical tissue as the phylogenetic scale progressed and the need for social survival increased. Obvious though the reasons for this may seem, it creates in humans a constant conflict which is apparent in the decision-making of everyday life. And here the reward system of the brain comes into play (RSB) ^1,2,11,15,24-27^.

The discovery and description of the RSB as well as its anatomical and neurochemical details are covered extensively in the literature^1,2,28-32^, and will not be reviewed in depth here. This system serves the manifold purposes of driving the animal to actively explore resources in the environment to satiate instinctual needs, selecting and developing motor behaviors toward prospective reward and away from prospective punishment, and also of signaling the actual results of the selected behavior with a positive or negative valence^11,24,26,27,33-37^. Malfunctions of the RSB may lead to anhedonia and lack of motivation, and imbalances in its function are key to drug abuse and other addictive behaviors^28,29,34,35,37-39^.

The RSB is mostly a subcortical circuit organized around the connections between the dopamine-producing mesencephalic nuclei (particularly the ventral tegmental area) and the nucleus accumbens. The latter, a striatal structure with dense connectivity to cortical and subcortical motor centers could be conceived as the structure providing the interface between motivation, and motor behavior toward the motivated behavior^30^. In line with other subcortical circuits, the RSB has been driving the behavior of mammals toward biologically relevant targets for millions of years and is thus well established as a major player in the equation determining behavior. Of major relevance to this paper is the fact that such an ancient system is solidly geared toward the consummation of behaviors leading to biologically (that is, survival) relevant gratifications, with a clear bias toward behaviors with a perspective of immediate or short-term rewards – congruent with their role in driving the satisfaction of instinctual needs^11,26,27,35,36,39^. The human link here is that any stimulus we receive from the environment or from our body or mind will be analyzed not only by the more modern, reason-related neocortical (particularly prefrontal) regions, but also by the relatively “inflexible” reward system which will always attempt to “co-opt” the behavioral response toward immediate or short term rewards. Thus, a constant conflict is always in play between this millions-of-year-old, ancient, “strong” system driving the behavior toward short-term rewarding consequences, and the much more recent, “less matured” prefrontal regions which will attempt to modulate this behavior directly influencing the activity of the RSB. Naturally, the end goal of the “social” prefrontal regions is to push the behavioral response toward less immediate and much longer-term advantageous social consequences. The problem is that the temporal dimension of these rewards – that is, time 2 – opposes the influences of the two systems. It is almost certain that most males, including of course humans, will have the drive to approach an attractive female, irrespective of social “constraints” such as those posed by marriage “combinations” (on either side) (1). However, in the vast majority of instances, a human male will respect the social combinations, deciding in the context of the more advantageous, longer term social consequences, and thus will not approach such a woman. A telling comparison would be with montane voles, small mammals unrestrained by social constraints and which simply do not maintain monogamous relationships^41^. In these, and the vast majority of mammals, the subcortical drive of the RSB clearly dominates the behavioral response equation.

A brief summary of this section is that when our human brains are functioning well, our reward system does signal the perspectives for immediate pleasure, but the prefrontal “filter”modulates this and ‘releases’ only those behaviors which are adequate for the specific social context (in time and space) of the individual. Therefore, the circumstances modulate the adequacy of a given behavior and thus the ‘healthy’, physiological predominance of the subcortical reward system, or of the prefrontal cortical system in each specific context. More specifically stated, when a pleasurable behavior leading to reward in the short term does not risk ‘social survival’, the prefrontal filter relaxes and the balance is skewed toward the predominance of the subcortical reward system. The opposite occurs when the long-term consequences will be negative and the same behavior needs to be suppressed. Of course, the “calibration” of this highly relevant prefrontal action in the response equation is fully dependent on the executive functions, particularly the ability to control impulses and condense present, past, and future through working memory mechanisms^6,10^.

## Summing up: the neurobiology of the gap between theory and practice

*Actual* behaviors toward stimuli depend upon the prefrontal cortex, its executive functions, and the ‘battle’ against the subcortical reward systems. However, *theorizing* about decisions is much less dependent upon the executive functions. The ‘intention-to-do’ something, reasoning about why this or that decision or behavior would be more appropriate deviates from the neurodynamics regulating actual behaviors in *practice*. Patients with severe prefrontal lesions may reason and theorize quite appropriately in regard to the best attitudes toward incoming stimuli. The crucial issue is that in practice, in real life, several stimuli – appealing differently to the subcortical reward and to the prefrontal systems - coexist in time. In other words, in practice, there are several stimuli with prospectively distinct levels of immediate versus delayed gratification demanding a behavioral response. The executive functions are actually needed to deal with these conflicting stimuli and to select the most appropriate behaviors. A practical example, borrowing from the analogy of a certain lady described above, may better illustrate this point.

Let us imagine that the young woman who wishes to lose weight is now, at 2 PM, after a satisfying lunch, having a consultation with a nutritional therapist. She is fully agreeing with the therapist’s advice regarding how important it is for her to control her eating habits and to refrain from many types of food. She gets somewhat euphoric with the prospect of losing weight, is sure that all the advice received makes perfect sense, and promises herself she will heed the advice. This is all very well, but has no real biological value in the sense that *theorizing about intentions* has no real link with final behavioral responses. The true challenge is reserved for the practical situation that will present itself when she is hungry a few hours later, and on the numerous other occasions during the many months ahead. In these practical moments of hunger she will have to decide between eating the delicious types of food she has been used to, versus refraining from those and eating only the less appetizing or reduced amounts prescribed by the nutritional therapist. Conflicting stimuli in this situation are easy to grasp and common to everyone: the smell and the view of the delicious ‘non-recommended’ dishes versus the internal motivation of controlling intake, losing weight, and getting healthier and more shapely for the coming Summer.In this very real situation, primed by her therapist, this woman will be inclined to decide based upon the delayed rewards of the hypothetical future, having however, to pay the price of dismissing the immediate pleasures at hand. It is at this very moment that the prefrontal cortical regions and their executive functions have to act! This gap between theory and practice reflects, indeed, the constant struggle of human behavior toward the environment. Overt pathology, the crafting of neural function through experience, and the genetic bases of temperament and personality all have an impact on how well a given person may bridge this gap^42^.

## An ‘avant-première’: the precedence of emotion in decision-making

In this essay we hope to have provided a background for decision-making processes in humans. However, the dynamics of the interaction between prefrontal ‘rational’ and subcortical ‘emotional’ systems in the final determination of behavior was only marginally touched upon. We believe this crucial aspect of human behavior could only be adequately discussed after dealing with the ‘basics’ of decision-making as we did here. As mentioned, we have reserved a full essay for this, in which we then hope to review the evidence pointing to a precedence of emotional mechanisms in the guidance of decisions in humans^43,44^. Even more, we plan to integrate the concepts of decision-making reviewed here with the different emotional styles we have as a richly heterogeneous species. Inescapably, the emotional tone and brilliance of all of us, unique individuals, drive us through the decisions we need to make in everyday life.

## Footnotes

* In a sense, this view of working memory as using past experiences to guide specific decisions in the present differs from the concept originally proposed by Baddeley. Classically, working memory was conceptualized as concerned with the ‘acute’ retention of recently acquired information just for the period of time needed to perform a *specific task*, after which the information could be discarded^13^. This mechanism would, almost by definition, bypass information processing through the hippocampus. The view presented here extends this concept by proposing that it is also within the realm of working memory to retrieve and retain for given periods of time previously acquired (and thus hippocampus-processed) information in the form of past experiences relevant for *specific decisions* in the present. Nevertheless, even if the type of retained information differs, a common denominator operates in both views, namely, the fact that the information has only to be retained *while a behavior is being performed* – and then can be ‘discarded from the surface’. Perhaps because of this conciliatory view, Baddeley has himself recently extended the concept of working memory constituents to include not only a central executive, a phonological loop and a visuospatial loop, but also an episodic buffer bridging past memories with present action through working memory mechanisms^14^.
